# Primary Cilia–Related Pathways Moderate the Development and Therapy Resistance of Glioblastoma

**DOI:** 10.3389/fonc.2021.718995

**Published:** 2021-08-26

**Authors:** Minghao Li, Jiaxun Zhang, Haonan Zhou, Rong Xiang

**Affiliations:** ^1^Xiangya School of Medicine, Central South University, Changsha, China; ^2^School of Life Sciences, Central South University, Changsha, China; ^3^Hunan Key Laboratory of Animal Models for Human Diseases, Central South University, Changsha, China

**Keywords:** glioblastoma, primary cilia, CCRK, LPAR1, HDAC6, PCM1, sonic hedgehog

## Abstract

As microtubule-based structures, primary cilia are typically present on the cells during the G0 or G1-S/G2 phase of the cell cycle and are closely related to the development of the central nervous system. The presence or absence of this special organelle may regulate the central nervous system tumorigenesis (e.g., glioblastoma) and several degenerative diseases. Additionally, the development of primary cilia can be regulated by several pathways. Conversely, primary cilia are able to regulate a few signaling transduction pathways. Therefore, development of the central nervous system tumors in conjunction with abnormal cilia can be regulated by up- or downregulation of the pathways related to cilia and ciliogenesis. Here, we review some pathways related to ciliogenesis and tumorigenesis, aiming to provide a potential target for developing new therapies at genetic and molecular levels.

## Introduction

### Glioblastoma

GBM is the most common and aggressive primary brain tumor in adults, accounting for 45.6% of primary malignant brain tumors, despite its low annual incidence of 3.1 per 100,000 compared with that of cancers deriving from other organs such as the breast and lung, with increased incidence and decreased survival rate with age (1). Majority of GBM patients suffer recurrence within 32–36 months, and median survival is approximately 10 months (2). Necrosis and endothelial proliferation are the defining histopathologic features of primary GBM, qualifying it as the highest grade of brain tumors in the World Health Organization (WHO) classification, i.e., grade IV. Secondary GBM refers to a minority of GBMs evolving from WHO grade III or IV gliomas (3). Clinical course of tissue destruction, edema, and epilepsy contribute to the clinical symptoms including personality changes, mood disorders, sensorimotor deficits, aphasia, and epilepsy (4, 5). Previous studies have suggested that adult GBM may be derived from neural stem and progenitor cells, which are located in the subventricular zone, subcortical white matter, and dentate gyrus of the hippocampus (6, 7). Standard treatment of GBM includes surgery, radiotherapy, antiangiogenic therapy, and alkylating chemotherapy with drugs such as temozolomide (TMZ). However, therapeutic efficacy is still limited despite rapid progress in cancer therapy. Consequently, there is a need for new therapeutic strategies for GBM. Thus, finding molecules and signaling pathways associated with proliferation, malignant development, and therapeutic resistance is significantly necessary to identify new targets for developing novel approaches for GBM treatment.

O6-methylguanyl DNA methyltransferase (MGMT), a DNA repair enzyme that counteracts the toxicity of TMZ, plays an important role in the proliferation and therapeutic resistance of GBM and remains the most significant biomarker in clinical decision making, especially in GBM recurrence (8, 9). As a downstream gene of the sonic hedgehog (SHH) pathway, whose activation depends on the presence of primary cilia, the expression level of MGMT is closely correlated with primary cilia and related pathways, accounting for the close relationship between GBM and primary cilia. Additionally, aberrant cilia have been found in six of seven biopsies of GBM patients, providing further evidence that primary cilia play an important role in the genesis and development of GBM (10).

### Primary Cilia

Cilia are microtubule-based organelles extending from the surface of cells, and they can be classified into motile and non-motile (primary) cilia (11). The latter are confirmed as highly conserved sensorial antenna that may detect several types of stimulations from the environment, growth factors, neurotransmitters, and even light (e.g., photoreceptor cells). This organelle is 1~10 μm long and 0.2~0.3 μm wide, and its core is an axoneme characterized as 9 + 0 ring structure composed of nine pairs of microtubule doublets surrounded by a bilayer lipid membrane in continuity with the plasma membrane (12). The cilia possess specific proteins and lipids that are required to maintain their bioactivity. The continuous region sinks in the cell body to form a structure called “ciliary pit” or “ciliary pocket,” which is closely related to endocytosis and formation of endosomes (13). Primary cilia usually exist in quiescent cells as basal bodies, which are modified centrioles, to initiate ciliogenesis, and they may be utilized to organize the mitotic spindle in cell division (11, 14). When the mitosis is complete, the mother centriole tends to transform into two distinct centrioles, but only one mature centriole is capable of nucleating a primary cilium at its distal end, and it carries fibrous appendages and satellites (15). To organize and maintain the organized cilia, the intraflagellar transport (IFT) is developed to transport the components used to form the axoneme to the ciliary tip *via* a bidirectional microtubule-based transportation system between the ciliary tip and base, which is operated by kinesin and dynein (16). Additionally, the primary cilia regulate the proliferation of other ciliated cells living in the microenvironment adjacent to the ciliated cell *via* ciliary tip excision and release of ciliary vesicles (10).

**Figure 1:**
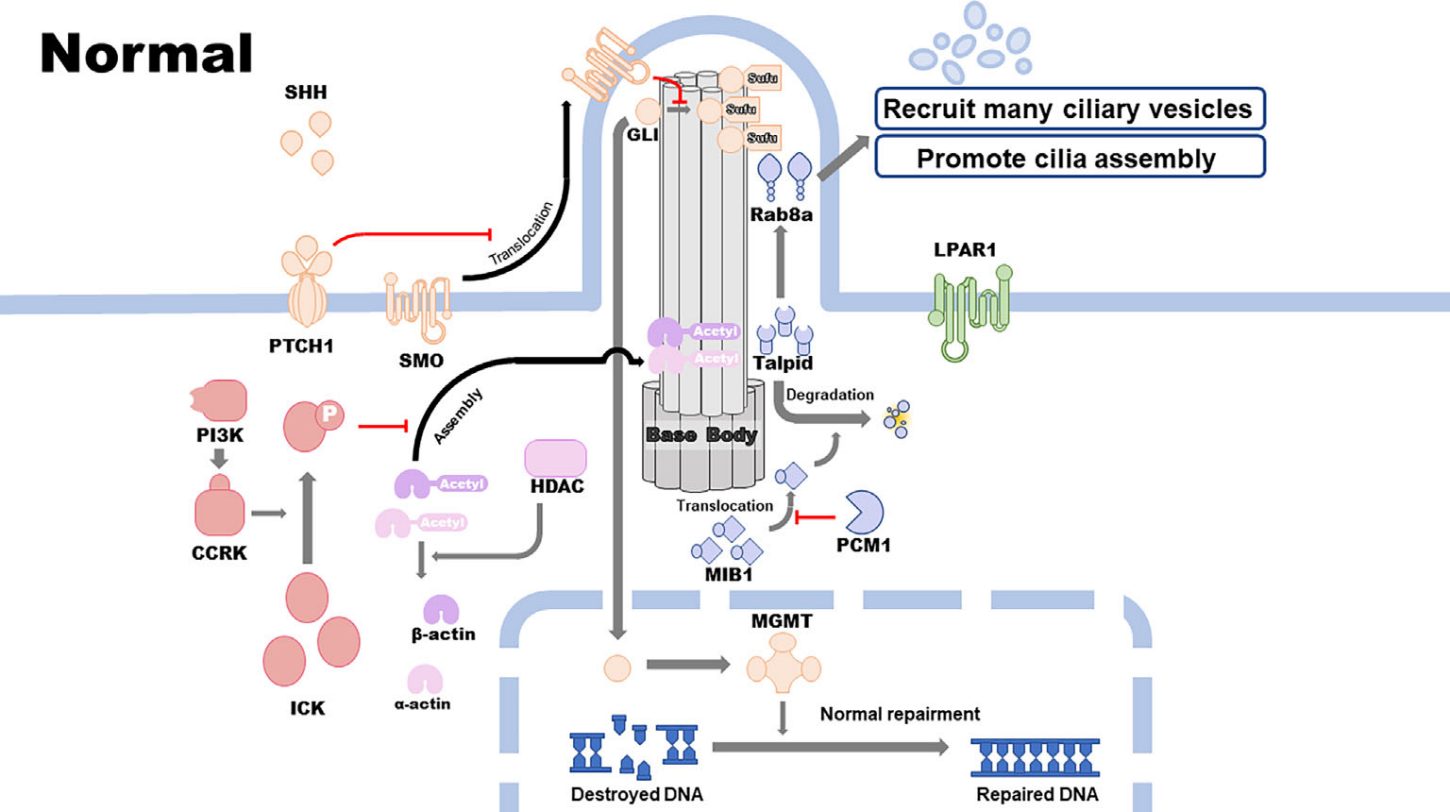
Cilia-related pathways in normal cells.

**Figure 2:**
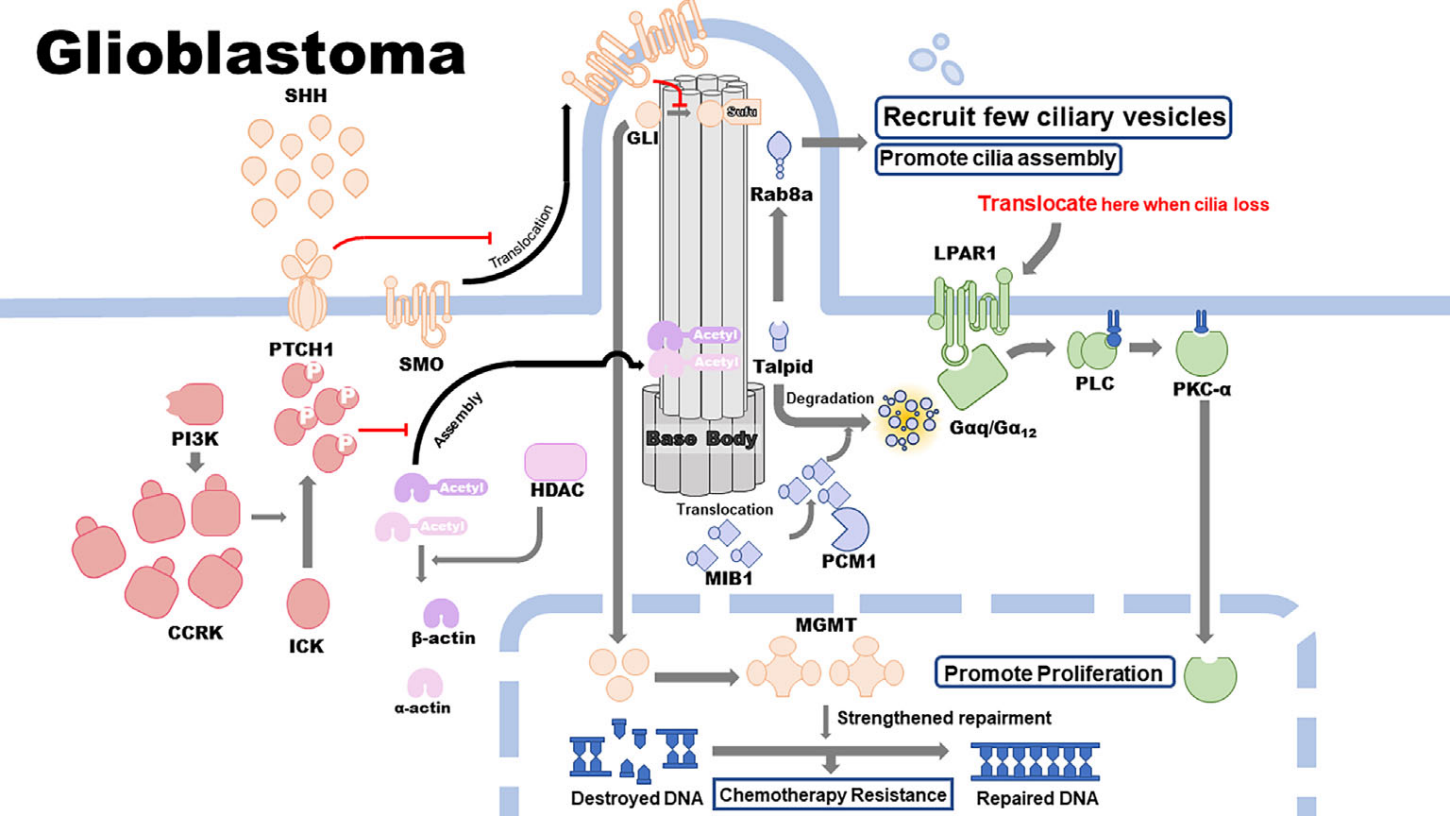
Cilia-related pathways in glioblastoma (GBM) cells.

Primary cilia are highly associated with GBM development and therapeutic resistance. Cilia-related signaling pathways, including the SHH, cell cycle–related kinase (CCRK), and histone deacetylase 6 (HDAC6) pathways, are closely correlated with the proliferation, malignant development, and therapeutic resistance of GBM. Other cilium-related pathways, including the lysophosphatidic acid receptor 1 (LPAR1) and pericentriolar material 1 (PCM1) pathways, inhibit the proliferation and development of GBM cell lines. Epidermal growth factor receptor (EGFR), alpha-type platelet-derived growth factor receptor (PDGFRɑ), MGMT, and isocitrate dehydrogenase 1 (IDH1) pathways promote GBM therapeutic resistance, which is associated with or modulated by the assembly and disassembly of primary cilia (**Figures 1** and **2**).

## Pathways Related to Primary Cilia and GBM

### The SHH Pathway

It is widely known that the SHH pathway is implicated in several physiologic and pathologic processes such as angiogenesis, development of the central nervous system (CNS), tumor proliferation, therapeutic drug resistance, and progression of some of the CNS tumors including GBM. Shh is one of the putative signaling molecules, implicated in the regulation of the CNS polarity and neural patterning (17). Shh is initially synthesized as a 45 kDa precursor protein, which is automatically cleaved into two secreted peptides, namely, 19 kDa Shh-N (amino terminus) and 26 kDa Shh-C (carboxy terminus). Shh-N has been shown to be the key location of the signal-mediating function of Shh, while Shh-C mediates its auto-proteolysis reaction. Auto-proteolysis results in the addition of a cholesterol molecule at the C-terminus of Shh-N and a palmitoyl group at the N-terminus of Shh, resulting in the formation of a dual lipid modified Shh-N, an activated form of Shh with increased inductive potency and better secretive regulation, transportation, and distribution ability, thereby enabling long-range SHH signaling (18–20).

The genes that regulate the presence or absence of cilia affect the activation of the SHH pathway, as the transduction and activation of this pathway require primary cilia as the structural base. It has been reported that Shh levels are much higher in the microenvironment of GBMs than in other tissues and that the activation of SHH signaling pathway requires the binding of Shh to the Patched (PTCH) mediated Smoothened (SMO) (PTCH-SMO) receptor complex (21), consequently resulting in a more activated SHH pathway in GBM. PTCH contains a sterol-sensing domain, which interacts with the cholesterol portion of the dual-lipid-modified Shh-N (21). PTCH is believed to suppress SMO activity directly and indirectly; however, when it binds to Shh, SMO is activated and stabilized, initiating the downstream signaling cascade by encoding membrane proteins, which are similar to G-protein-coupled receptors (22). While primary cilia exist, the signal transduction process tends to be initiated by the binding of Shh protein in the extracellular matrix to PTCH1 located on the membrane surrounding the axoneme of the primary cilia. The binding between these two molecules triggers PTCH1 removal from the primary cilia, followed by translocation of SMO from the adjacent membrane to the cilia, where it inhibits the transformation of the active form of Gli transcription factor family (GLIA) into the repressor form, consequently leading to the accumulation of GLIA. GLI, Cos2, Fu, and suppressor of fused (Sufu) form a tetrameric complex for the downstream signaling process. This complex results in the transcription activity of GLI. Cos2 is a kinesin-like protein, which is mainly a motor domain that binds to ATP and microtubules (23). Fu is a segment polarity protein, which binds to Cos2 *via* its carboxy-terminal and is phosphorylated in response to hedgehog signaling (24). Sufu is a negative regulator of the SHH signaling pathway. When Shh ligand is not present, Sufu directly binds to GLI and inhibits its translocation to the nucleus, resulting in the cleavage of GLI by proteasomes into transcriptional repressors, thereby preventing the downstream pathway activation. Various molecules in the Gli transcription factor family display different functions, such as regulation of the transcription of several oncogenes and tumor-suppressor genes, modulation of tumor progression, and development of resistance to some therapeutic drugs (25). For instance, MGMT, a DNA repair enzyme that can repair damage in cancer cell DNA induced by some alkylating agents (e.g., TMZ), contributes significantly to the development of resistance to therapeutic drugs specific to target cell DNA (e.g., TMZ) (26). Moreover, MGMT is considered a downstream gene whose transcript level can be regulated by GLI1 (26, 27), a member of Gli transcription factor family. When the expression of genes upstream of the SHH pathway is upregulated, the active form of GLI1 leads to its accumulation and translocation to the nucleus, and then, it upregulates the expression of MGMT at the transcription level. The increased expression of MGMT results in enhanced repair of destroyed DNA, which in turn leads to development of resistance to TMZ. A recent study revealed that acquired kinase inhibitor resistance is associated with upregulation of ciliogenesis, failure of control of cilia length, and increased activation of the SHH pathway. Manipulating cilia length and integrity *via* Kif7 knockdown can resensitize GBM cells and confer drug resistance (28). Collectively, the SHH pathway, the activation of which depends on primary cilia, can regulate the development of malignant phenotype and resistance towards therapeutic drugs *via* downstream transcription factors, which can regulate several oncogenes and tumor-suppressor genes.

### The LPAR1 Signaling Pathway

Lysophosphatidic acid (LPA), which exists widely in the human body, is a small bioactive glycophospholipid extracted from membrane phospholipids with many different biological functions associated with GBM progression, such as stimulation of cell proliferation, cytoskeleton recombination, cell survival, cell differentiation, and DNA synthesis and ion transport, *via* binding to a variety of G-protein-coupled receptors (9). LPAR1 acts as the central signaling hub for extracellular-intracellular and intracellular-extracellular signal transduction, mediating various molecular and biological functions, including cell survival, cell migration, cell adhesion, cytoskeletal changes, cell proliferation, immune function, Ca2^+^ mobilization, and myelination (29, 30).

LPAR1 is associated with GBM, and it has been confirmed that the primary cilia can moderate GBM development and cell proliferation *in vivo* and *in vitro via* the LPAR1 signaling pathway (31). A recent study clearly demonstrated the interaction between cilia and LPAR1 signaling pathway in GBM cells (31). When cilia are present, LPAR1 is sequestered in the primary cilia, and compartmentalization of LPAR1 and its downstream effectors, Gɑ12 and Gɑq, which are located on the cell membrane, prevents the interaction between LPAR1 and G-protein-coupled receptor subunits, restricting its cell proliferative signaling. In contrast, loss of the primary cilia eliminates spatial barriers and results in redistribution of LPAR1 to the plasma membrane, where it binds to Gɑ12 and Gɑq and facilitates the activation of downstream pathways, promoting the proliferative function of LPA and unlocking the potential for unlimited proliferation of GBM cells, consequently maintaining a highly proliferative phenotype. LPAR1 inhibitor Ki16425 reduces cell growth only in deciliated astrocytes, thereby validating the abovementioned findings. Furthermore, Gɑq activates phospholipase C, which produces diacylglycerol (DAG) from phosphatidylinositol 4,5-bisphosphate. DAG activates classic and novel protein kinase C (PKC) (32). Valdés-Rives et al. conducted *in vitro* experiments and found that LPAR1 induces PKC ɑ isozyme (PKCɑ) translocation from cell membrane to the nucleus in GBM cells *via* G-protein-coupled receptors, promoting GBM cell growth (33). Taken together, the loss of cilia can induce LPAR1 relocation and activate downstream G-protein-coupled receptor signaling pathways, enhancing the proliferation of GBM cells. This is suggestive of a potential target site of GBM prevention and treatment.

### The CCRK-ICK/MAK Pathway

CCRK overexpression is found in conjunction with loss of cilia among various CNS tumors, including GBM, and the high expression level is typically in concert with the high mortality and poor prognosis of patients with CNS tumors. Additionally, homologs of CCRK, such as LF2 in *Chlamydomonas* and NIH3T3 in zebrafish, can regulate the formation and length of primary cilia (34, 35). Collectively, these findings suggest that CCRK may play a key role in the human primary cilia formation, subsequently contributing to the occurrence and development of several tumors. Yang et al. indicated that overexpression of CCRK, whose upstream regulator is phosphoinositide 3-kinase, can induce loss of cilia through its substrate ICK/MAK, consequently promoting the proliferation of GBM cells (36). Overexpression of CCRK leads to a loss of cilia induced by serum starvation *via* phosphorylation activation of ICK, resulting in a reduced ability to inhibit cell cycle re-entry of cilia and deregulation of the cell cycle progression, as determined *via* EdU incorporation (37). The majority of cilia loss is induced by the activation of ICK, which is caused by the kinase activity of CCRK, and the rest is caused by the structure of non-phosphorylated ICK. Therefore, CCRK is considered as an oncogene owing to its ability to induce cell cycle progression and increase the proliferative capacity of GBM cells. Accordingly, depletion of CCRK and ICK can inhibit the proliferation of cancer cells by stabilizing the structure and function of the primary cilia in a KIF3a-dependent manner (35). In addition, the primary cilia–dependent SHH pathway is also regulated by CCRK, as CCRK possesses the ability to inhibit ciliogenesis. As an early event in the activation of the SHH pathway, translocation of Gli3 is significantly upregulated following depletion of CCRK (36).

### The HDAC6-Related Pathway

As an epigenetic modification, acetylation induced by histone acetyltransferase (HAT) can reduce the expression level of target genes. HDAC6 was initially found to be a deacetylase that can remove an acetyl group from the histone and, subsequently, restore the expression level of genes downregulated by acetylation (38). Therefore, the balance between HAT and HDAC6 levels plays an important role in the maintenance of normal expression of genes encoding structural or non-structural proteins, including some oncogenes and tumor-suppressor genes, and consequently, this balance maintains the homeostasis in the human body and prevents the occurrence and development of some tumors. When the balance between HAT and HDAC6 levels is dysregulated, the probability of tumor occurrence significantly increases due to an imbalance in the expression of oncogenes and tumor-suppressor genes. Apart from histone deacetylation, HDAC6 can also deacetylate some proteins located in the plasma or on the cell membrane, such as acetylated α-tubulin (27). As the assembly of the primary cilia requires acetylated α-tubulin, HDAC6 may deacetylate and destroy the protein component needed to form the axoneme of the primary cilia. Therefore, high expression of HDAC6 tends to appear in concert with the loss of the primary cilia in GBM biopsies and cultured cell lines. Taken together, *HDAC6* has been gradually considered as an oncogene as its overexpression may promote malignancy and enhanced proliferation *via* inhibition of assembly of the primary cilia. Thus, depletion of HDAC6 levels, which results in promotion of ciliogenensis as well as cell apoptosis induced by TMZ and reversal of malignancy, is becoming a potential strategy in developing new therapies for GBM. In addition to the reversion of malignancy induced by HDAC6 depletion, alteration in the sensitivity of GBM cells to TMZ is triggered by not only HDAC6 depletion but also deactivation of the SHH pathway due to the loss of cilia, leading to a reduction in the MGMT level and DNA repairing ability (39, 40).

### The PCM1-Regulated Pathway

PCM1 is an essential pericentriolar protein that plays a significant role in cell division and survival, especially in centrosome stability, microtubule and actin organization, and centriolar duplication prior to mitosis and ciliogenesis, in normal and malignant cells, such as GBM cells (41). Centriolar satellites, majorly composed of PCM1, are non-membrane cytoplasmic granules that are located around the centrosome and play crucial roles in transporting centrosome proteins from the cytoplasm to the centrosome during centrosome assembly or ciliogenesis. Therefore, diseases, such as ciliopathies, may possibly occur when centriolar satellites become deficient (42). In addition, PCM1 has been shown to bind to several centrosome proteins to ensure that they are accurately located (43, 44). Thus, PCM1, centriolar satellites, and the centrosome are significantly implicated, closely correlated, and function together in cell cycle dynamics and ciliogenesis in GBM cells.

As a key component of centriolar satellites and the functional activity of the centrosome, PCM1 plays a vital role in GBM cell mitosis, cell cycle, cell proliferation, and sensitivity to standard-of-care therapeutic agent TMZ *via* regulation of ciliogenesis (41). PCM1 restricts Mindbomb 1, an E3 ligase associated with Notch signaling, to satellites *via* tethering, and thus, prevents its translocation to centrioles, which would otherwise promote Talpid3 destabilization through polyubiquitination, failure to recruit ciliary vesicles associated with Talpid3-bianding protein Rab8a, or inhibition of cilium assembly (45, 46). PCM1-associated cilia formation thus inhibits GBM progression and cell proliferation. Furthermore, studies have found that sorting nexin 17, a P-selectin binding protein, can recruit and stabilize ubiquitin-specific protease 9X, a deubiquitinating enzyme that is able to regulate the integrity of centriolar satellites in mitotic cells, antagonize Mindbomb 1-induced ubiquitination and degradation of PCM1 *via* binding to PCM1, and inhibit PCM1 ubiquitination during ciliogenesis (47, 48). In addition, Plk4, a conserved protein kinase that regulates the centriole duplication cycle, is required for PCM1 phosphorylation, followed by adequate spatial distribution and organization of centriolar satellites, which facilitate ciliogenesis (49, 50). By sequestering proteins in the centriolar satellite compartment, PCM1 promotes ciliogenesis, thereby inhibiting GBM progression.

## Pathways Related to Cilia and Therapy Resistance of GBM Subtypes

Based on genomic and transcriptomic data, the Cancer Genome Atlas Research Network (2008) has provided a detailed view of the genomic alterations and affected signaling pathways and stratified GBM into four subtypes: classical, mesenchymal, proneural, and neural GBM (51). Each subtype displays different genomic features that affect the resistance mechanisms associated with or modulated by the primary cilia (**Table 1**).

**Table 1:** Therapeutic strategy and cilia-related pathways associated with glioblastoma development and therapy resistance.

Cilia-related pathway		Promotion/inhibition of proliferation	Therapeutic strategy
Pathway involved in GBM development	SHH	Promote	Vismodegib (52), Sonidegib (52)
	LPAR1	Inhibit	ND
	CCRK-ICK/MAK	Promote	ND
	HDAC6	Promote	Ricolinostat (53), Citarinostat (53)
	PCM1	Inhibit	ND
Pathway involved in GBM therapy resistance	EGFR	Promote	Afatinib (54), Dacomitinib (55), Panitumumab (56, 57)
	PDGFRɑ	Promote	SHP099 (58)
	MGMT	Promote	TMZ (59)

GBM, glioblastoma; SHH, sonic hedgehog; LPAR1, lysophosphatidic acid receptor 1; CCRK, cell cycle-related kinase; HDAC6, histone deacetylase 6; PCM1, pericentriolar material 1; EGFR, epidermal growth factor receptor; PDGFRɑ, alpha-type platelet-derived growth factor receptor; MGMT, O6-methylguanyl DNA methyltransferase; TMZ, temozolomide; ND, not determined.

### Classical GBM

Classical GBM is defined by *EGFR* amplification and *CDKN2* depletion with a distinct lack of additional abnormalities in *TP53*, *NF1*, *PDGFRɑ*, or *IDH1* genes (60). EGFR extensively regulates cellular processes during cell development, and it is critically associated with tumorigenesis and tumor cell invasion (61). EGFR has been found to be localized on the primary cilia in astrocytes and neuroblasts (62). EGFR activates phosphatidylinositol (3,4,5)-trisphosphate, partly through phosphoinositide 3-kinase-induced phosphorylation of phosphatidylinositol 4,5-bisphosphate into phosphatidylinositol (3,4,5)-trisphosphate and the hydrolysis of phosphatidylinositol 4,5-bisphosphate by activation of phospholipase C-gamma 2 (63). Following this, phosphatidylinositol (3,4,5)-trisphosphate activates the Akt signaling pathway and regulates the cell cycle (64). Amplified EGFR expression in classical GBM can sustain EGFR signaling, Akt activity, and DNA repair mechanisms *via* the primary cilia and, thus, promote resistance towards EGFR-targeted therapy (65). In addition, classical GBM has the highest MGMT methylation rate among the four subtypes, which confers a strong ability of repairing destroyed DNA and, hence, resistance to TMZ therapy (66).

### Mesenchymal GBM

Mesenchymal GBM is characterized by a high frequency of *NF1* mutation/deletion, low *NF1* mRNA expression, and *PTEN* mutation (60). Studies have shown that a loss of *NF1* expression results in resistance to endocrine therapy, through both estrogen receptor-dependent and -independent mechanisms in advanced breast cancer (67). However, associations between NF1 activity and cilia-related therapeutic resistance in mesenchymal GBM remain unclear.

### Proneural GBM

Proneural GBM is characterized by amplification of *PDGFRɑ* expression and point mutations in *IDH1* and *TP53* genes (60). PDGFRα localizes to the primary cilia in astrocytes and neuroblasts. PDGFRα receptor in the primary cilium leads to the onset of a number of downstream signaling pathways within the cilium, including the Akt and Mek1/2-Erk1/2 pathways, to regulate the cell cycle and directional cell migration (64). *IDH1* mutations have previously been associated with secondary GBM (68). Hence, amplification of PDGFRα expression can sustain PDGFRα signaling *via* the primary cilia and enhance resistance of proneural GBM to therapy.

### Neural GBM

Neural GBM is genetically associated with neural, astrocytic, and oligodendrocytic gene signatures. Their expression patterns are similar to those in the normal brain tissue, which can be differentiated based on the morphology using light microscopy (60).

## Conclusion

GBM is the most common and malignant brain tumor in adults with poor prognoses, short recurrence period, and low survival rate. Common cancer therapies, such as surgical resection, radiotherapy, and chemotherapy, are widely applied for treating GBM, but the therapeutic effect remains to be limited. Therefore, there is a need to explore new therapeutic strategies to combat GBM. Studies have shown that the primary cilia may play dual roles in GBM progression, which should be highly considered for devising potential methods of GBM treatment. Thus, cilia-elated signaling pathways could be novel targets for GBM treatment.

In this review, we summarized five primary cilium-related signaling pathways moderating the development and therapeutic resistance of GBM. The SHH pathway promotes DNA repair and TMZ resistance. Drugs targeting the SHH pathway have been developed, of which vismodegib and sonidegib have been approved by the Food and Drug Administration for treating basal cell carcinoma (52). Thus, it is necessary to develop SHH inhibitors for GBM treatment, which is possible. The CCRK-ICK/MAK pathway induces cilium loss and promotes GBM development. Drugs targeting CCRK are being developed, but no CCRK-specific inhibitor is publicly available due to lacking information on the 3D structure of the CCRK protein (69). Accordingly, research on the 3D structure of the CCRK protein is essential to develop CCRK inhibitors for GBM therapy. HDAC6 inhibits cilia formation, and HDAC6 inhibitors ricolinostat and citarinostat are currently being tested for cancer treatment (53). Therefore, it is necessary to test the efficacy of these HDAC6 inhibitors and conduct clinical trials for use in GBM treatment. The PCM1 pathway facilitates cilia formation and inhibits GBM progression, while the LPAR1 pathway promotes GBM cell proliferation. Considering that no drugs targeting these two pathways have been reported, it is possible and suggestive to develop new GBM therapies targeting the PCM1 and LPAR1 pathways.

In conclusion, further research is required to obtain a deeper understanding of the potential involvement of and the signaling pathways related to the primary cilia in regulating cell proliferation and the impact on GBM development and its resistance to therapeutic drugs. We expect major progress in the following years, which may allow for designing cilia-based and molecular targeted therapies to offer new treatment strategies against GBM.

## Author Contributions

ML wrote the manuscript and took charge of the figures. JZ researched the subject and wrote the manuscript. HZ was responsible for the figures. Embellishing the article was conducted by RX. All authors contributed to the article and approved the submitted version.

## Funding

This study was supported by the National Science and Technology Major Project of the Ministry of Science and Technology of China (2017ZX10103005-006), the National Natural Science Foundation of China (81970403).

## Conflict of Interest

The authors declare that the research was conducted in the absence of any commercial or financial relationships that could be construed as a potential conflict of interest.

## Publisher’s Note

All claims expressed in this article are solely those of the authors and do not necessarily represent those of their affiliated organizations, or those of the publisher, the editors and the reviewers. Any product that may be evaluated in this article, or claim that may be made by its manufacturer, is not guaranteed or endorsed by the publisher.
